# DeeWaNA: An Unsupervised Network Representation Learning Framework Integrating Deepwalk and Neighborhood Aggregation for Node Classification

**DOI:** 10.3390/e27030322

**Published:** 2025-03-20

**Authors:** Xin Xu, Xinya Lu, Jianan Wang

**Affiliations:** 1 School of Media Science, Northeast Normal University, Jingye Street 2555, Changchun 130117, China; xux894@nenu.edu.cn; 2 School of Journalism, Northeast Normal University, Jingye Street 2555, Changchun 130117, China; 3 School of Information Science and Technology, Northeast Normal University, Jingye Street 2555, Changchun 130117, China; luxy111@nenu.edu.cn; 4 School of Physics, Northeast Normal University, Renmin Street 5268, Changchun 130024, China

**Keywords:** unsupervised network representation learning, node classification, random walk, neighborhood aggregation, graph embedding

## Abstract

This paper introduces DeeWaNA, an unsupervised network representation learning framework that unifies random walk strategies and neighborhood aggregation mechanisms to improve node classification performance. Unlike existing methods that treat these two paradigms separately, our approach integrates them into a cohesive model, addressing limitations in structural feature extraction and neighborhood relationship modeling. DeeWaNA first leverages DeepWalk to capture global structural information and then employs an attention-based weighting mechanism to refine neighborhood relationships through a novel distance metric. Finally, a weighted aggregation operator fuses these representations into a unified low-dimensional space. By bridging the gap between random-walk-based and neural-network-based techniques, our framework enhances representation quality and improves classification accuracy. Extensive evaluations on real-world networks demonstrate that DeeWaNA outperforms four widely used unsupervised network representation learning methods, underscoring its effectiveness and broader applicability.

## 1. Introduction

Network Representation Learning (NRL) aims to learn latent, low-dimensional network representational vectors [1]. With information networks becoming ubiquitous in describing the complex relationships in large real-world applications in the forms of social networks, biological networks, and citation networks, NRL has played a crucial role in many network analysis applications, including node classification, link prediction, community detection, and subnetwork similarity [1,2,3,4]. Because of the advantages of adaption for the absence of expensive manual annotation data, many Unsupervised Network Representation Learning (UNRL) methods [5,6,7,8,9,10] have been proposed and have shown remarkable achievements.

DeepWalk is one of the widely used random-walk-based UNRL algorithms, due to its few-shot friendliness and simple distributed computation. It is based on context sampling of node sequences on networks to calculate representation vectors, which results in a reduced capacity for capturing neighborhood messages. Many researches have demonstrated the importance of catching neighborhood messages in network representation learning, and various neighborhood aggregation methods have been proposed [11,12,13,14]. Integrating DeepWalk with neighborhood aggregation techniques appears to be a promising strategy that provides representational vectors with improved performance for machine learning models in downstream network analysis tasks.

**Figure 1:**
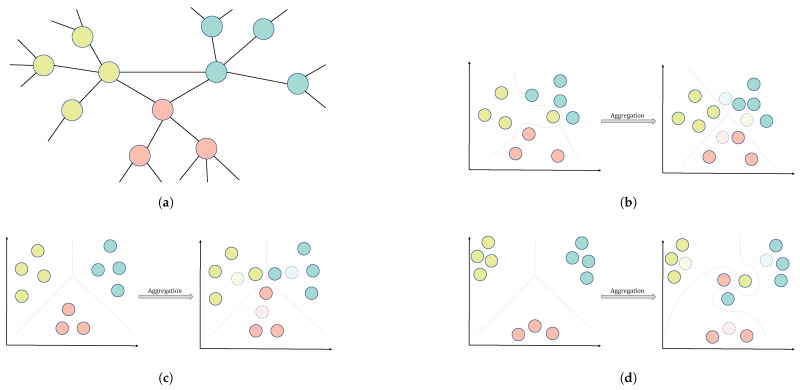
An example of node classification updated by different neighborhood aggregation operators: (**a**) Network G. (**b**) Neighborhood aggregation techniques can reduce difficulty of the classification task. (**c**) Poor neighborhood aggregation techniques will arrange neighbor nodes closely together in the embedding space. (**d**) Catastrophic aggregation techniques make tasks more difficult.

Although the existing aggregation methods differ from model to model [15], the distances between nodes and their neighbors in embedding space are not taken into account by the approaches. The updated representational vectors may cause tasks to become more challenging when neighborhood nodes that are located at varying distances from a central node are considered equally throughout neighborhood aggregation operators. We illustrate an example of a node classification task, as shown in Figure 1. Given a network *G*, as shown in Figure 1a, neighborhood aggregation techniques can reduce the difficulty of the classification task from the perspective of position in the embedding space, as shown in Figure 1b. In certain circumstances, poor aggregation techniques will arrange neighbor nodes closely together in the embedding space, as shown in Figure 1c. In some awful circumstances, catastrophic aggregation techniques might make tasks more difficult, as shown in Figure 1d. As a result, the neighbors should be distinguished by distinct weights throughout the aggregation process. Many works have used the attention mechanism, a weighting technique, to discriminate between neighbors [10,16].

In this paper, we presented the DeeWaNA framework for unsupervised network representation learning, which combines DeepWalk with neighborhood aggregation. Specifically, DeeWaNA initially makes use of DeepWalk to obtain the nodes’ self-representational vectors. Then, the framework employs a weighted neighborhood aggregation operation to produce local representational vectors of nodes based on their self-representational vectors. To distinguish between the various effects of neighbor nodes on their center node, DeeWaNA uses the self-attention between nodes and their one-hop neighbor nodes as the weights in the weighted neighborhood aggregation process. For the purpose of obtaining self-attention, we suggest a Euclidean distance-based learning task on representational vectors in embedding space. DeeWaNA fuses the sequential connecting self-representational vectors and local representational vectors into a single unified embedding space using the Principal Component Analysis (PCA) method [17].

We conducted thorough tests to evaluate DeWaNA against four widely used unsupervised network representation learning algorithms on a range of four real-world datasets for the single-label/multi-label node classification tasks. Our experiments demonstrate that DeeWaNA can provide the network representational with enhanced performance to machine learning models on single-label/multi-label node classification tasks.

## 2. Related Work

It is common practice to employ random-walk-based approaches for unsupervised network representation learning [18,19]. Deepwalk is based on a strategy of choosing neighbors with equal probability for sampling node sequences that are fed into the skip-gram model for network embedding [6]. The node2vec algorithm is an improved version of DeepWalk, which utilizes two parameters for adjusting the usage of a deep-first search and a breadth-first search in sampling node sequences [9]. The off-the-shelf random-walk-based approach, however, is unable to fully utilize the neighbors’ message that is frequently employed in deep-structure-based network representation learning models.

Many deep-structure-based models have been proposed for network representation learning [5,11,12]. Neighborhood aggregation plays an important role in neural-network-based models for learning network representational vectors with high performance [5,13,15,20,21,22]. These studies do not take into account the various effects of neighbors on aggregation operators. By utilizing masked self-attentional stacking layers, which may implicitly assign various weights to different nodes in a neighborhood without expensive matrix operation or knowing graph topology beforehand, Graph Attention Networks (GATs) [10] are utilized to attend to the impacts of neighbors. Although GATs can discriminate between various neighbors, they need labels to acquire self-attention meaning; hence, designing learning tasks is necessary when employing GATs in unsupervised network representation learning.

## 3. Methodology

In this section, we offer a framework called DeeWaNA that combines DeepWalk with weighted neighborhood aggregation for unsupervised network representation learning. DeeWaNA incorporates weighted neighborhood aggregation into DeepWalk to improve previous random-walk-based unsupervised network representation learning techniques that are unable to collect neighbor information. Consequently, by utilizing DeepWalk and the weighted neighborhood aggregation technique to gather neighborhood information, DeeWaNA can produce high-performance network representation.

### 3.1. The General Framework of DeeWaNA

**Figure 2:**
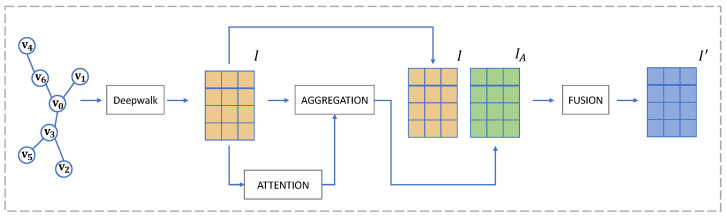
Framework overview. First, DeepWalk is utilized to obtain self-representational vectors of nodes. Then, the weights between neighbors are calculated by the self-attention model on a Euclidean-distance-based learning task. The local representational vectors of nodes are calculated by the weighted neighborhood aggregation. Finally, the sequentially connected vectors are fused into a single unified embedding space.

We first present the general framework of DeeWaNA, which incorporates DeepWalk and weighted neighborhood aggregation techniques and is illustrated in Figure 2.

First, given a network *G*, where *V* is the set of nodes and *E* is the set of edges, we use DeepWalk, defined as f(G), to obtain the representational vectors of nodes in *V*. The $r_{v},v\in V$ is the self-representational vector of node *v*, which is calculated as follows:

(1) $$\left\{r_{0},r_{1},\ldots ,r_{v}\right\}=f\left(G\right);$$

Then, the nodes’ self-representational vectors are utilized to calculate the Euclidean distance between the nodes and their neighbors in embedding space, which serves as the aim of learning self-attention. By using weighted neighborhood aggregation, the local representational vectors of nodes are created from their neighbors’ self-representational vectors. Finally, the sequentially connected vectors of the self-representational vectors and the local representational vectors are fused into a single unified embedding space by Principle Component Analysis (PCA).

### 3.2. Weighted Neighborhood Aggregation

The process of neighborhood aggregation is frequently used to collect neighborhood information from adjacent nodes. The neighborhood information of node *u* is embedded as the local representational vector $r_{u}^{\prime }$. The $r_{u}^{\prime }$ is calculated as follows:

(2) $$r_{u}^{\prime }=\frac{1}{\left|n(u)\right|}\sum_{v\in n(u)}r_{v},$$

where n(u) is the set of the neighbors of the node *u*, and $r_{v}$ is the self-representational vector of node *v*. DeeWaNA calculates the weights between nodes and incorporates the weights into the neighborhood aggregation to distinguish the influences of neighbor nodes on the central node. The local representational vector of node *u*, using weighted neighborhood aggregation, is calculated as follows:

(3) $$r_{u}^{\prime }=\sum_{v\in n(u)}\alpha_{uv}*r_{v};$$

where n(u) represents the set of neighbors of node *u*, $\alpha_{uv}$ is the weight between node *u* and node *v*, and $r_{v}$ is the self-representational vector of node *v*. The next section presents three distinct weighting calculations.

### 3.3. Euclidean-Distance-Based Weighting Calculations

Self-attention is a widely used learning attention method to weight the various relationship between nodes and their neighbors [23]. To obtain the weights between nodes and their neighbors, we proposed three Euclidean-distance-based weighting calculations.

#### 3.3.1. One-Head Self-Attention

**Figure 3:**
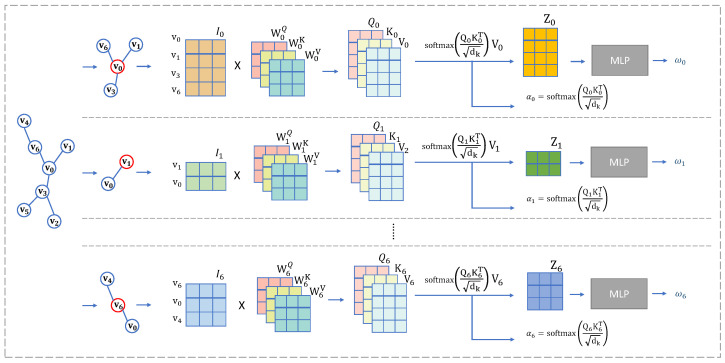
Framework of one-head self-attention: Each one of lines calculates the weights of one node’s neighbors. The top line is the process of calculation for the weights of neighbors of node $v_{0}$, the middle line is for $v_{1}$, and so on.

The self-representational vectors of nodes and the vectors of their neighbors are used as inputs in the one-head self-attention model to determine the attention (weights), as shown in Figure 3. The attention function is described as follows:

(4) $$Attention\left(Q,K,V\right)=softmax\left(\frac{QK^{T}}{\sqrt{d_{k}}}\right)V$$

where $Q=I*W^{Q}$, $K=I*W^{K}$, and $V=I*W^{V}$; $I\in R^{n*d_{model}}$ is the matrix of representation, the learning matrices are $W^{Q}\in R^{d_{model}*d_{k}},W^{K}\in R^{d_{model}*d_{k}},W^{V}\in R^{d_{model}*d_{v}}$, n=|I| is the size of inputs, and $d_{model}$ is the dimension of representation. The outputs are put into a full-connect Multi-Layer Perception (MLP). The MLP consists of three layers with ($n*d_{model},\frac{n*d_{model}}{2},n-1$) neurons. The estimated weights of node *i* by one-head self-attention are calculated as follows:

(5) $$\alpha_{i}=softmax\left(\frac{Q_{i}K_{i}^{T}}{\sqrt{d_{k}}}\right).$$

We utilize the distance in embedding space *d* between nodes as the target of the learning task. The output of the MLP is the estimated distance $d^{\prime }$. We aim to minimize the loss function as follows:

(6) $$Loss\left(d^{\prime },d\right)=\sqrt{{\left(d^{\prime }-d\right)}^{2}}$$

for training the self-attention model.

#### 3.3.2. Multi-Head Self-Attention

**Figure 4:**
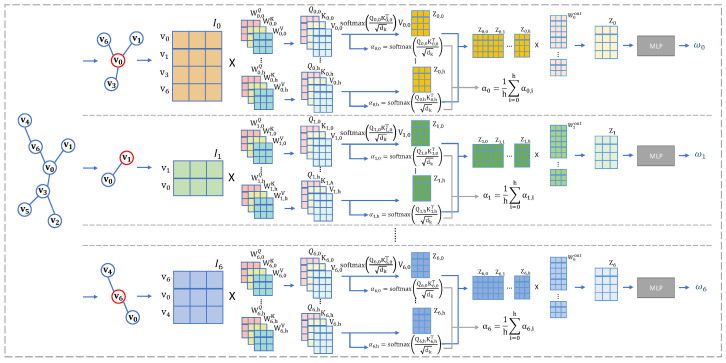
The multi-head self-attention mechanism employs parallel computation units to independently derive attention weights for different nodes in the graph structure. Specifically, each attention head generates a distinct set of learnable projection parameters that computes the scaled dot-product attention between a target node and its neighbors. As illustrated in the computational graph, the uppermost attention head processes the edge weight relationships for node $v_{0}$, and the intermediate head handles the neighborhood interactions for node $v_{1}$, with this pattern extending to subsequent nodes. Crucially, all attention heads operate concurrently through parameter-independent transformation matrices, enabling the model to capture heterogeneous relational patterns in the feature space.

In contrast to the one-head self-attention model with queries (Q), keys (K), and values (V), multi-head self-attention attends to information from various representational subspaces, as depicted in Figure 4. Each subspace of representation is processed by the one-head self-attention function. The calculation of multi-head self-attention is described as follows:

(7) $$MultiHead{\left(I\right)}_{\theta }=Splice\left(head_{1},\ldots ,head_{h}\right)W^{out}$$

where $head_{i}=Ateention\left(IW_{i}^{Q},IW_{i}^{K},IW_{i}^{V}\right)$, $\theta =\{W_{1\ldots h}^{Q},W_{1\ldots h}^{K},W_{1\ldots h}^{V},W^{out}\}$, $W^{Q}\in R^{d_{model}*d_{k}},$$W^{K}\in R^{d_{model}*d_{k}},$$W^{V}\in R^{d_{model}*d_{v}},W^{out}\in R^{hd_{v}*d_{model}}$. Each subrepresentation is calculated by Equation (5). The value of the multi-head self-attention is calculated as follows:

(8) $$\alpha =\frac{1}{h}\sum_{i=0}^{h}\alpha_{i}$$

For the MLP structure, there are three layers with ($n*d_{model}$, $\frac{n*d_{model}}{2}$,n−1) neurons (n=|I|).

#### 3.3.3. Non-Learning Weighting Calculation

**Figure 5:**
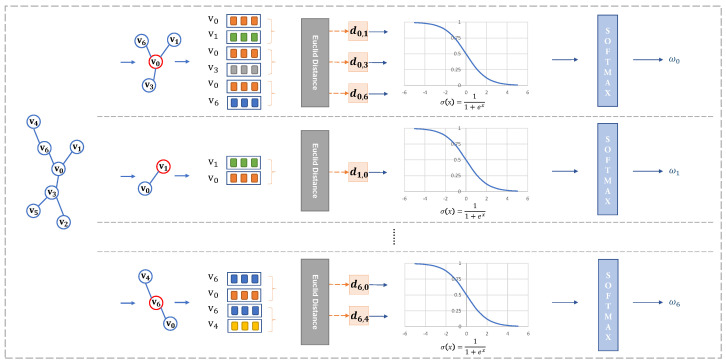
Non-learning weighting. From left to right: calculating the Euclidean distance between nodes and their neighbors; normalizing the distance into (0,1); through the Softmax function, obtaining the weights.

Self-attention demands unacceptably long computation times and memory requisition on some large networks. To solve this issue, a straightforward weighting calculation is suggested and illustrated in Figure 5. The distance $d_{uv}$ between node *u* and node *v* is normalized into the (0, 1) range as follows:

(9) $$d_{uv}^{\prime }=\frac{1}{1+e^{d_{uv}}}$$

The weight between node *v* and node *u* is calculated by the following:

(10) $$w_{uv}=\frac{e^{d_{uv}^{\prime }}}{\sum_{d_{uj}^{\prime }}e^{d_{uj}^{\prime }}},j\in \left\{n\left(u\right)\right\};$$

where n(u) is the set of neighbors of node *u*. The non-learning approach substitutes the self-attentional method to calculate the weights between nodes when a node has a large number of neighbors.

### 3.4. Connection and Fusion

DeeWaNA fuses the linked representational vectors into a single unified embedding space by PCA after splicing the local representational vectors after the self-representational vectors. This is because the simple connection of two different-view representational vectors may cause adverse effects on following network analysis tasks due to the representational vectors coming from different domains.

## 4. Experiments

We evaluated DeeWaNA on four real-world networks and compared it against four widely used unsupervised network representation learning algorithms, including (**DeepWalk** [6], **LINE** [7], **Node2vec** [9], and **SDNE** [8]). We set the dimension of representation in all the methods as 128 and used the two-step representation in the LINE. The restricted number of neighbors was set to 10 in all simplified versions. We set the number of heads in multi-head self-attention to 4. Other parameters were set as default in corresponding papers. All experiments were run on the platform of 2.8 GHz 4-core Intel i7, 16 GB 2133 MHz LPDDR3.

### 4.1. Datasets

We employed four real-world datasets (**Blogcatalog** [24], **Protein-Protein Interactions (PPI)** [25], **Amherst, Mich** [26]) to evaluate the performance of DeeWaNA, and the detailed statistics of these datasets are listed in Table 1.

### 4.2. Experimental Results

We utilized a one-vs-rest logistic regression model [6] to evaluate the quality of the learned representational vectors of all methods in node classification tasks. The training set was created by randomly selecting a proportion of the nodes, and the testing set was created using the remaining nodes. We repeated this process 10 times to calculate the average values on the Micro-F1.

#### 4.2.1. Results on Multi-Label Classification Tasks

For all multi-label networks, DeeWaNA steadily performed better than other competitors within the rate of training data from 0.2 to 0.8. The experimental results of the multi-label classification tasks on Blogcatalog and PPI are displayed in Table 2 and Table 3.

On the Blogcatalog dataset, DeeWaNA with the non-learning weighting calculation produced the best performance (bold indicated) across all training proportions. One-head/multi-head self-attention was unable to produce the desired results due to memory limitations, as indicated by ‘-’. On the PPI dataset, DeeWaNA performed best at training proportions of (0.2, 0.8) when using the non-learning weighting calculation and at (0.4, 0.6) when using a combination of the multi-head self-attention and the non-learning calculation.

#### 4.2.2. Results on Single-Label Classification Tasks

For all single-label networks, DeeWaNA consistently outperformed its competitors in the 0.2 to 0.8 range of training data proportions. The experimental results of single-label node classification tasks on Amherst and Mich are displayed in Table 4 and Table 5. When one-head self-attention and the non-learning calculation were coupled, DeeWaNA fared the best (bold indicated) in all training proportions on the Amherst dataset. On the Mich dataset, when utilizing the one-head self-attention and the non-learning calculation, DeeWaNA scored best at the training proportions of (0.4, 0.6, and 0.8) and at (0.2) when using the multi-head self-attention and the non-learning weighting calculation.

Our findings show that the various weighting calculations for DeeWaNA perform differently in the multi-label networks, while the combination of the one-head self-attention and the non-learning weighting calculation performs well in the majority of situations in the single-label networks. In single-label tasks, we advise utilizing one-head self-attention along with a non-learning weighting calculation, and in multi-label tasks, choose an appropriate weighting calculation.

#### 4.2.3. Study on Effectiveness of Weighted Neighborhood Aggregation

To demonstrate the effectiveness of the weighted neighborhood aggregation, we conducted an experimental investigation applying weighted neighborhood aggregation to three widely used unsupervised network representation learning methods. LiNA is the technique that uses LINE instead of DeepWalk in DeeWaNA. NoNA is the method that substitutes the node2vec algorithm for DeepWalk in DeeWaNA. SeNA is the approach that replaces DeepWalk in DeeWaNA with the SDNE method. In this part, all methods utilized non-learning weighting calculation. The experimental results are displayed in Table 6.

The experimental results showed that, with the exception of the LINE algorithm on PPI and the node2vec method on Amherst at the 0.2 training proportion, the weighted neighborhood aggregation could improve the corresponding unsupervised learning algorithm in all cases. We speculate that the neighborhood aggregation amplifies the noise in the poor-quality self-representational vectors obtained by node2vec and SDNE in the low training proportion. In addition, comparing DeeWaNA with LiNA, NoNA, and SeNA, as described in Table 7, applying DeepWalk in DeeAWaNA was the best option for obtaining self-representational vectors for all cases except on the Mich dataset. Even though NoNA outperformed other algorithms on the Mich dataset in terms of the (0.2, 0.4, 0.8) training proportions, we still favor DeepWalk due to the node2vec’s good performance in NoNA depending on hyper-parameter tuning.

#### 4.2.4. Study on Relationship Between Distance and Weights

To explore the relationship between distance and weights, we compared two kinds of non-learning weighting calculations in DeeWaNA on multi-/single-label classification tasks. DeeWaNA processed the weights being inversely proportional to distances. The calculation is as defined in Equation (9). DeeWaNA (F) processed the weights being proportional to distances. In DeeWaNA (F), the distance $d_{uv}$ between node *u* and node *v* is normalized into the (0, 1) range as follows:

(11) $$d_{uv}^{\prime }=\frac{1}{1+e^{-d_{uv}}}$$

All DeeWaNA versions employed the non-learning weighting calculation in this part. The experimental results are displayed in Table 8. We found that the DeeWaNA (N) obtained the dominant position in all cases. This is also in line with our intuition that the farther the distance, the less the influence.

#### 4.2.5. Study on Effectiveness of Features

We carried out an experimental investigation with two DeeWaNA variants on the Blogcatalog dataset to demonstrate the effectiveness of the local representational vectors. DeeWaNA (local) applied the local representational vectors as the final representations. DeeWaNA (cnt) regarded the unfused connection of self-representational vectors and local representational vectors as the final representations. All DeeWaNA versions employed the non-learning weighting calculation in this investigation. The experimental results are listed in Table 9. Comparing the ‘DeepWalk’ and the ‘DeeWaNA (local)’ results, the local-representational vectors have less expressive ability than the self-representational vectors. Comparing the ‘DeepWalk’ and the ‘DeeWaNA (cnt)’ results, the unfused connected representational vectors are more expressive than the self-representational vectors. Comparing the ‘DeeWaNA (cnt)’ and the ‘DeeWaNA (N)’ results, the performance of representation could be enhanced by fusing the linked representational vectors into a single unified embedding space using principal component analysis. The ablation experiment demonstrated the effectiveness of local representational vectors as assistants and the usefulness of the fusion process.

#### 4.2.6. Study on Fusion

We compared DeeWaNA against two variants of DeeWaNA, which are DeeWaNA (AE) and DeeWaNA (ICA). In this part, all of DeeWaNA versions applied non-learning weighting calculation. The DeeWaNA (AE) consists of a five-layer (256-192-128-192-256) encoder–decoder neural network model as the fusion part. The DeeWaNA (ICA) used the Independent Component Analysis algorithm [27] as the fusion part. The experimental results are displayed in Table 10, and the best results are shown in bold. The results demonstrate that the PCA as the fusion process in DeeWaNA could obtain the best performance.

#### 4.2.7. Study on Multi-Head Self-Attention

**Figure 6:**
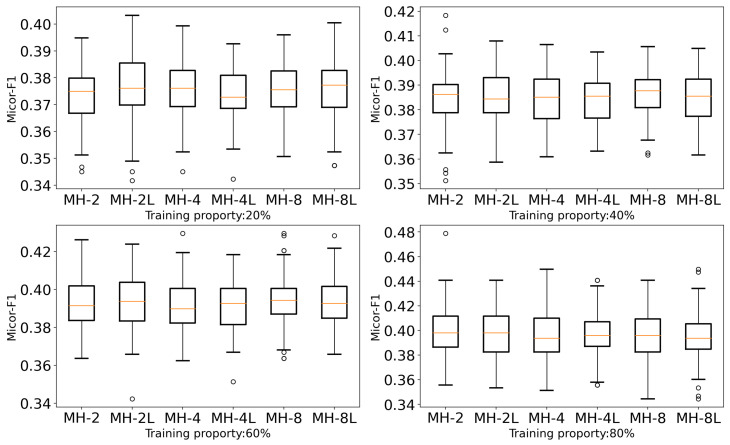
The comparison of different heads of multi-head self-attention on the Amherst dataset.

We investigated the 2/4/8 heads (MH-2/4/8) and the combined multi-head self-attention 2/4/8 heads with non-learning weighting calculations (MH-2L/4L/8L) to ascertain the number of heads in the multi-head self-attention models. The experimental results are displayed in Figure 6. Despite the fact that no particular number of heads predominated over others in all training proportions of the multi-head self-attention models, through the comparison of the stability and the worst case of prediction, we set the multi-head to four in this paper.

### 4.3. Limitations

Overall, the DeeWaNA could effectively obtain network representational vectors with good performance on the single-label and multi-label node classification tasks. The weighted neighborhood aggregation can easily be applied to improve some unsupervised network representation learning methods. But, there are some limitations in this work. First, for simplified calculations, only one-hop neighbors’ impacts were considered when DeeWaNA processed weighted neighborhood aggregation. The loss of ignoring multi-hop aggregation will be discussed in future works. Second, some high time-consuming or high-memory-required unsupervised network representation learning methods are not included in this work due to our hardware performance limitations. Third, more research is required before claiming weighted neighborhood aggregation can be applied generally, because this work lacks some SOTA unsupervised network representation learning methods that are particularly time- or memory-intensive. We think that despite many limitations, it is still a good attempt to give existing non-deep-structure-based unsupervised representation learning algorithms the ability to capture neighbor information.

## 5. Conclusions

In this study, we propose DeeWaNA, an unsupervised network representation learning framework that synergistically integrates DeepWalk with neighborhood-aware aggregation for enhanced node classification. The framework’s effectiveness stems from three key innovations: First, DeeWaNA enhances the conventional DeepWalk approach by incorporating neighborhood information awareness, thereby improving the discriminative power of learned node representations. Second, we introduce an adaptive weighted aggregation mechanism that dynamically quantifies heterogeneous neighbor influences during feature integration. Third, we develop a geometry-aware learning paradigm using Euclidean-distance-based self-attention to preserve spatial relationships in the embedding space.

Through comprehensive experimental evaluation on multiple real-world datasets across different domains, we demonstrate DeeWaNA’s superior performance against four unsupervised network representation learning baselines. The consistent outperformance across evaluation metrics reveals that DeeWaNA generates more semantically meaningful representations that significantly boost downstream node classification accuracy compared to existing approaches.

## Figures and Tables

**Table 1:** Statistics of datasets.

Dataset	$\left|V\right|$	$\left|E\right|$	Multi	# Labels
Blogcatalog	10,312	333,983	Yes	39
PPI	3890	76,584	Yes	50
Amherst	2235	90,954	No	6
Mich	3745	81,901	No	10

**Table 2:** Experimental results of multi-label classification on Blogcatalog on Micro-F1 (%). Bold indicates the optimal result among the comparative algorithms under the same parameters.

Algorithm	Training Proportion			
	0.2	0.4	0.6	0.8
DeepWalk	38.49	40.67	41.78	42.51
LINE	29.66	33.74	35.54	36.76
node2vec	37.99	39.69	40.72	41.33
SDNE	29.62	30.45	30.75	30.95
DeeWaNA (N)	**39.44**	**41.27**	**42.25**	**42.91**
DeeWaNA (1H + N)	39.36	41.23	42.11	42.83
DeeWaNA (MH + N)	39.37	41.25	42.19	42.72
DeeWaNA (1H)	-	-	-	-
DeeWaNA (MH)	-	-	-	-

**Table 3:** Experimental results of multi-label classification on PPI on Micro-F1 (%). Bold indicates the optimal result among the comparative algorithms under the same parameters.

Algorithm	Training Proportion			
	0.2	0.4	0.6	0.8
DeepWalk	18.30	20.58	22.01	22.88
LINE	12.29	14.04	15.44	16.69
node2vec	18.23	20.37	21.66	22.72
SDNE	16.81	18.47	19.34	20.08
DeeWaNA (N)	**19.99**	22.44	23.67	**24.55**
DeeWaNA (1H + N)	19.91	22.37	23.68	24.54
DeeWaNA (MH + N)	19.85	**22.52**	**23.70**	24.47
DeeWaNA (1H)	19.96	22.39	23.57	24.54
DeeWaNA (MH)	19.97	22.37	23.68	24.47

**Table 4:** Experimental results of single-label classification on Amherst on Micro-F1 (%). Bold indicates the optimal result among the comparative algorithms under the same parameters.

Algorithm	Training Proportion			
	0.2	0.4	0.6	0.8
DeepWalk	35.61	37.27	38.58	39.60
LINE	31.60	34.38	36.52	37.63
node2vec	36.38	37.18	37.96	38.53
SDNE	35.42	36.88	37.89	38.88
DeeWaNA (N)	37.53	38.66	39.38	39.98
DeeWaNA (1H + N)	**37.64**	**38.74**	**39.59**	**40.13**
DeeWaNA (MH + N)	37.41	38.39	39.11	39.68
DeeWaNA (1H)	37.54	38.45	39.05	39.56
DeeWaNA (MH)	37.53	38.43	39.13	39.62

**Table 5:** Experimental results of single-label classification on Mich on Micro-F1 (%). Bold indicates the optimal result among the comparative algorithms under the same parameters.

Algorithm	Training Proportion			
	0.2	0.4	0.6	0.8
DeepWalk	39.70	42.16	43.68	44.53
LINE	35.33	38.11	39.89	41.33
node2vec	39.40	42.06	43.44	44.20
SDNE	36.46	38.38	39.60	40.01
DeeWaNA (N)	39.62	42.11	43.70	44.64
DeeWaNA (1H + N)	39.60	**42.20**	**43.72**	**44.78**
DeeWaNA (MH + N)	**39.80**	42.19	43.53	44.70
DeeWaNA (1H)	39.49	41.95	43.47	44.48
DeeWaNA (MH)	39.65	42.01	43.29	44.69

**Table 6:** The experimental results of effectiveness of weighted neighborhood aggregation on Micro-F1 (%). Bold indicates the optimal result among the comparative algorithms under the same parameters.

Dataset	Algorithm	Training Proportion			
		0.2	0.4	0.6	0.8
Blogcatalog	LINE	29.66	33.74	35.54	36.76
	LiNA	**34.58**	**36.60**	**37.71**	**38.31**
	node2vec	37.99	39.69	40.72	41.33
	NoNA	**38.85**	**40.36**	**41.17**	**41.67**
	SDNE	**29.62**	30.45	30.75	30.95
	SeNA	29.21	**30.70**	**31.55**	**32.08**
PPI	LINE	12.29	14.04	15.44	16.69
	LiNA	**16.70**	**18.39**	**19.52**	**20.18**
	node2vec	18.23	20.37	21.66	22.72
	NoNA	**19.94**	**22.00**	**23.07**	**23.78**
	SDNE	16.81	18.47	19.35	20.08
	SeNA	**17.86**	**20.20**	**21.51**	**22.31**
Amherst	LINE	31.60	34.38	36.52	37.63
	LiNA	36.34	37.61	38.62	39.31
	node2vec	**36.38**	37.18	37.96	38.53
	NoNA	36.37	**37.48**	**38.11**	**38.93**
	SDNE	35.42	36.88	37.89	38.88
	SeNA	**37.36**	**38.54**	**39.15**	**39.77**
Mich	LINE	35.33	38.11	39.89	41.33
	LiNA	**38.21**	**40.47**	**41.64**	**42.57**
	node2vec	39.40	42.06	43.44	44.20
	NoNA	**39.84**	**42.38**	**43.64**	**44.80**
	SDNE	36.46	38.38	39.60	40.01
	SeNA	**37.61**	**39.34**	**40.25**	**40.78**

**Table 7:** The experimental results of various algorithms for learning self-representational vectors on Micro-F1 (%). Bold indicates the optimal result among the comparative algorithms under the same parameters.

Dataset	Algorithm	Training Proportion			
		0.2	0.4	0.6	0.8
Blocatalog	LiNA	34.58	36.60	37.71	38.31
	NoNA	38.85	40.36	41.17	41.67
	SeNA	29.21	30.70	31.55	32.08
	De*NA (N)	**39.36**	**41.27**	**42.25**	**42.91**
PPI	LiNA	16.70	18.39	19.52	20.18
	NoNA	19.94	22.00	23.07	23.78
	SeNA	17.86	20.20	21.51	22.31
	De*NA (N)	**19.99**	**22.44**	**23.67**	**24.55**
Amherst	LiNA	36.34	37.61	38.62	39.31
	NoNA	36.37	37.48	38.11	38.93
	SeNA	37.36	38.54	39.15	39.77
	De*NA (N)	**37.53**	**38.66**	**39.38**	**39.98**
Mich	LiNA	38.21	40.47	41.64	42.57
	NoNA	**39.84**	**42.38**	43.64	**44.80**
	SeNA	37.61	39.34	40.25	40.78
	De*NA (N)	39.62	42.11	**43.70**	44.64

**Table 8:** The classification results of different distance–weight relationships on Micro-F1 (%). Bold indicates the optimal result among the comparative algorithms under the same parameters.

Dataset	Algorithm	Training Proportion			
		0.2	0.4	0.6	0.8
Blogcatalog	DeeWaNA (F)	39.36	41.26	42.14	42.74
	DeeWaNA (N)	**39.44**	**41.27**	**42.25**	**42.91**
PPI	DeeWaNA (F)	19.87	22.42	23.62	24.54
	DeeWaNA (N)	**19.99**	**22.44**	**23.67**	**24.55**
Amherst	DeeWaNA (F)	37.44	38.48	39.23	39.88
	DeeWaNA (N)	**37.53**	**38.66**	**39.38**	**39.98**
Mich	DeeWaNA (F)	39.60	42.08	43.61	44.51
	DeeWaNA (N)	**39.62**	**42.11**	**43.70**	**44.64**

**Table 9:** The experimental results of various combinations of representation on Blogcatalog on Micro-F1 (%). Bold indicates the optimal result among the comparative algorithms under the same parameters.

Algorithm	Training Proportion			
	0.2	0.4	0.6	0.8
DeepWalk	38.49	40.67	41.78	42.51
DeeWaNA (local)	36.12	38.60	39.67	40.32
DeeWaNA (cnt)	38.68	40.89	41.96	42.67
DeeWaNA (N)	**39.44**	**41.27**	**42.25**	**42.91**

**Table 10:** The classification results of different fusion methods on Blogcatalog on Micro-F1 (%). Bold indicates the optimal result among the comparative algorithms under the same parameters.

Algorithm	Training Proportion			
	0.2	0.4	0.6	0.8
DeeWaNA (ICA)	17.64	18.61	19.81	21.12
DeeWaNA (AE)	35.21	37.62	38.73	39.45
DeeWaNA (N)	**39.36**	**41.27**	**42.25**	**42.91**

## Data Availability

Data are contained within the article.

## References

[1] Daokun Z., Jie Y., Xingquan Z., Chengqi Z. (2017). Network Representation Learning: A Survey. IEEE Trans. Big Data.

[2] Cannistraci C.V., Alanis-Lobato G., Ravasi T. (2013). Minimum curvilinearity to enhance topological prediction of protein interactions by network embedding. Bioinformatics.

[3] Tu C., Wang H., Zeng X., Liu Z., Sun M. (2016). Community-enhanced network representation learning for network analysis. arXiv.

[4] Xu X., Fu Y., Xiong H., Jin B., Li X., Hu S., Yin M. (2018). Dr. right!: Embedding-based adaptively-weighted mixture multi-classification model for finding right doctors with healthcare experience data. Proceedings of the 2018 IEEE International Conference on Data Mining (ICDM).

[5] Scarselli F., Gori M., Tsoi A.C., Hagenbuchner M., Monfardini G. (2008). The graph neural network model. IEEE Trans. Neural Netw..

[6] Perozzi B., Al-Rfou R., Skiena S. Deepwalk: Online learning of social representations. Proceedings of the 20th ACM SIGKDD International Conference on Knowledge Discovery and Data Mining.

[7] Tang J., Qu M., Wang M., Zhang M., Yan J., Mei Q. Line: Large-scale information network embedding. Proceedings of the 24th International Conference on World Wide Web.

[8] Wang D., Cui P., Zhu W. Structural deep network embedding. Proceedings of the 22nd ACM SIGKDD International Conference on Knowledge Discovery and Data Mining.

[9] Grover A., Leskovec J. node2vec: Scalable feature learning for networks. Proceedings of the 22nd ACM SIGKDD International Conference on Knowledge Discovery and Data Mining.

[10] Veličković P., Cucurull G., Casanova A., Romero A., Lio P., Bengio Y. (2017). Graph attention networks. arXiv.

[11] Kipf T.N., Welling M. (2016). Semi-supervised classification with graph convolutional networks. arXiv.

[12] Hamilton W., Ying Z., Leskovec J. Inductive representation learning on large graphs. Proceedings of the Advances in Neural Information Processing Systems.

[13] Hu F., Zhu Y., Wu S., Huang W., Wang L., Tan T. (2021). GraphAIR: Graph representation learning with neighborhood aggregation and interaction. Pattern Recognit..

[14] Sun Z., Wang C., Hu W., Chen M., Qu Y. Knowledge Graph Alignment Network with Gated Multi-Hop Neighborhood Aggregation. Proceedings of the AAAI Conference on Artificial Intelligence.

[15] Xie Y., Li S., Yang C., Wong C.W., Han J. When Do GNNs Work: Understanding and Improving Neighborhood Aggregation. Proceedings of the Twenty-Ninth International Joint Conference on Artificial Intelligence and Seventeenth Pacific Rim International Conference on Artificial Intelligence IJCAI-PRICAI-20.

[16] Wang Y., Sun Y., Liu Z., Sarma S.E., Bronstein M.M., Solomon J.M. (2018). Dynamic Graph CNN for Learning on Point Clouds. ACM Trans. Graph..

[17] Tipping M.E., Bishop C.M. (1999). Probabilistic principal component analysis. J. R. Stat. Soc. Ser. B (Stat. Methodol.).

[18] Zhang Y., Tang M. (2021). Consistency of random-walk based network embedding algorithms. arXiv.

[19] Guo K., Zhao Z., Yu Z., Guo W., Lin R., Tang Y., Wu L. (2022). Network Embedding Based on Biased Random Walk for Community Detection in Attributed Networks. IEEE Trans. Comput. Soc. Syst..

[20] Gallicchio C., Micheli A. Graph Echo State Networks. Proceedings of the 2010 International Joint Conference on Neural Networks (IJCNN).

[21] Li Y., Tarlow D., Brockschmidt M., Zemel R. (2017). Gated Graph Sequence Neural Networks. arXiv.

[22] Shen Y., Li H., Li D., Zheng J., Wang W. (2022). ANGraph: Attribute-interactive neighborhood-aggregative graph representation learning. Neural Comput. Appl..

[23] Vaswani A., Shazeer N., Parmar N., Uszkoreit J., Jones L., Gomez N.A., Kaiser L., Polosukhin I. Attention Is All You Need. Proceedings of the Advances in Neural Information Processing Systems 30 (NIPS 2017).

[24] Tang L., Liu H. (2009). Relational learning via latent social dimensions. Proceedings of the 15th ACM SIGKDD International Conference on Knowledge Discovery and Data Mining.

[25] Stark C., Breitkreutz B.J., Chatr-Aryamontri A., Boucher L., Oughtred R., Livstone M.S., Nixon J., Van Auken K., Wang X., Shi X. (2010). The BioGRID interaction database: 2011 update. Nucleic Acids Res..

[26] Traud A.L., Mucha P.J., Porter M.A. (2012). Social structure of facebook networks. Phys. A Stat. Mech. Its Appl..

[27] Hyvärinen A., Oja E. (2000). Independent component analysis: Algorithms and applications. Neural Netw..

